# Simultaneous Determination of Caffeic Acid and Ferulic Acid Using a Carbon Nanofiber-Based Screen-Printed Sensor

**DOI:** 10.3390/s22134689

**Published:** 2022-06-21

**Authors:** Alexandra Virginia Bounegru, Constantin Apetrei

**Affiliations:** Department of Chemistry, Physics and Environment, Faculty of Sciences and Environment, “Dunărea de Jos” University of Galaţi, 47 Domnească Street, 800008 Galati, Romania; alexandra.meresescu@ugal.ro

**Keywords:** hydroxycinnamic acid, cyclic voltammetry, carbon nanofibers, phyto-homeopathic product

## Abstract

This work aims to achieve the simultaneous qualitative and quantitative determination of two hydroxycinnamic acids (ferulic acid and caffeic acid) from standard solutions and from a phyto-homeopathic product using a carbon nanofiber-based screen-printed sensor (CNF/SPE). The two compounds are mentioned in the manufacturer’s specifications but without indicating their concentrations. The stability and reproducibility of the CNF/SPE were found to be effective and the sensitivity was high for both caffeic acid—CA (limit of detection 2.39 × 10^−7^ M) and ferrulic acid—FA (limit of detection 2.33 × 10^−7^ M). The antioxidant capacity of the compounds in the analyzed product was also determined by the DPPH (2,2-diphenyl-1-picrylhydrazyl) method. The electrochemical method was efficient and less expensive than other analytical methods; therefore, its use can be extended for the detection of these phenolic compounds in various dietary supplements or pharmaceutical products.

## 1. Introduction

Food supplements are formulations that contain micro- or macronutrients, together with excipients, intended to be consumed in various concentrations to maintain the body’s health [1]. Hydroxycinnamic acids such as caffeic acid and ferulic acid are often found in the composition of nutritional supplements, in particular for their antioxidant [2,3,4], hypoglycemic [5,6], anti-inflammatory [7,8] and hepatoprotective [9,10] roles. The health benefits of phenolic compounds depend on the amount consumed, but also on their bioavailability [11]. For example, in the US, daily consumption of phenolic compounds ranges from 200 mg^−1^ g [12]. Due to the fact that most dietary supplements and phytopreparations contain several active ingredients with variable and diverse polyphenol content, there is a need to develop simple and rapid methods for the simultaneous detection of these compounds. Such methods would also be extremely useful in the screening of different plants, in industry or in the evaluation or monitoring of food quality.

Techniques such as high-performance liquid chromatography coupled with chemiluminescence [13], UV-Vis [14], liquid chromatography in tandem with mass spectrometry [15,16] or coulometric methods [17] are methods often employed for the quantitative determination of polyphenols in various plant products, plant preparations or foodstuffs.

Although the efficiency of these techniques is well known, a disadvantage can be the long analysis time, the use of large amounts of solvents and samples, and the higher cost. In addition, spectrophotometric methods used individually cannot differentially analyze certain phenolic compounds. Therefore, it would be necessary to combine UV-Vis spectrophotometric detection, for example, with separation by liquid chromatography or capillary electrophoresis [18,19], a procedure that complicates and delays the results.

Therefore, there is a need for the development of rapid and less expensive analytical methods that allow the simultaneous quantification and evaluation of several polyphenolic compounds present in dietary supplements and phytopreparations.

Electrochemical detection techniques may be an optimal solution due to their simple operating steps, lower cost, portable instruments and the possibility to use them in practical applications in various fields [20]. These methods allow simultaneous but individual detection of analytes in a mixture without the need for a separation method [12].

Several modified electrodes have been described in the literature for the simultaneous determination of phenolic compounds. Some of them are glassy carbon electrodes modified with graphite, carbon nitride (C_3_N_4_) and chitosan [21], sensors based on multilayer carbon nanotubes and a surfactant [22], electrodes based on graphite paste modified with poly(dopamine quinone-chromium (III) complex) microspheres [23] or a glassy carbon electrode modified with a carboxymethyl-botriosferan polysaccharide and nanostructured black carbon [24].

As dietary supplements and phytopreparations contain many biologically active compounds, the electrochemical signals obtained are highly complex and the interpretation of the results can be difficult.

The use of a glassy carbon electrode [25] or a multilayer carbon nanotube-based electrode [26] has been reported in the literature, where strong and irreversible adsorption of phenolic compounds on the electrode surface has been observed. Thus, the detection peak was modified, obtaining multiple signals.

Such limitations make research on the selective determination of phenolic compounds a constant topic of interest. The selection of nanomaterials used in sensor development can make a difference, thus improving the results obtained [27]. To our knowledge, there are no studies aiming at the simultaneous determination of caffeic and ferulic acid by cyclic voltammetry; therefore, our study can be a starting point for further research in the simultaneous quantification of these polyphenols from different types of samples.

In the present study, a carbon nanofiber-based screen-printed electrode (CNF/SPE) was used for the simultaneous quantification of two hydroxycinnamic acids (caffeic acid and ferulic acid) from a product with a complex phyto-homeopathic formula, with a role in reducing the occurrence of visual impairment. CNF/SPE was chosen on the basis of previous studies, in which it demonstrated high sensitivity and reproducibility [28,29,30,31]. Additionally, in this study, CNF/SPE was found to be suitable and effective with a low detection limit for the determination of both electroactive compounds. The electrochemical technique used was cyclic voltammetry.

The study also aimed to determine the antioxidant capacity of these compounds in the Eye Blend product by the DPPH (common abbreviation for the organic chemical compound 2,2-diphenyl-1-picrylhydrazyl) method.

## 2. Materials and Methods

### 2.1. Reagents and Solutions Used

For the preparation of the phosphate buffer solution (0.1 M pH 7.0), NaH_2_PO_4_ and Na_2_HPO_4_ (Sigma-Aldrich, St. Louis, MO, USA) of analytical grade and ultrapure water obtained using a Millipore Milli-Q system were used. The pH was measured using a pH meter (WTW, Weilheim, Germany).

A 10^−3^ M potassium ferrocyanide solution, purchased from Sigma-Aldrich (St. Louis, MO, USA), was used for preliminary analyses.

Stock solutions (10^−3^ M) were prepared by dissolving ferulic acid and caffeic acid, respectively, of analytical purity, in 10^−1^ M PBS solution, pH 7.0. Both compounds were purchased from Sigma-Aldrich.

The stock solutions were stored at 4 °C and in the absence of light until use.

2,2-Diphenyl-1-picrylhydrazyl, DPPH, was purchased from Sigma-Aldrich (St. Louis, MO, USA) and the chloroform for dilutions and sample preparation from Chemical Company (Iasi, Romania).

### 2.2. Equipment and Determinations

All voltammetric analyses were performed using an EG&G potentiostat/galvanostat (Princeton Applied Research, Oak Ridge, TN, USA), model 263A, controlled by ECHEM software. The electrochemical cell (Princeton Applied Research, Oak Ridge, TN, USA), had a volume of 50 mL. The reference electrode used was Ag/AgCl and the auxiliary electrode was a platinum wire. A carbon nanofiber screen-printed electrode (CNF/SPE) purchased from Metrohm DropSens (Oviedo, Spain) was used as the working electrode. The electroanalytical method used was cyclic voltammetry. The experimental conditions were optimized and set at −0.4 and +1.3 V for the analyses performed in caffeic acid and ferulic acid solutions. In the case of potassium ferrocyanide, the optimal potential range was between −0.6 and +1.0 V.

Validation of the voltammetric method was performed using the DPPH method (determination of antioxidant activity). The method is based on the decolorization of 2,2-diphenyl-1-picrylhydrazyl radical (DPPH), colored purple [32].

2,2-diphenyl-1-picrylhydrazyl (DPPH) is a stable free radical as it contains an unpaired electron, and the molecule does not dimerize unlike other free radicals because the free electron of DPPH is delocalized. The DPPH solution in chloroform is violet in color with maximum absorbance at 520 nm [33]. The combination of DPPH and a molecular species capable of donating hydrogen results in a reduction process, where DPPH loses its purple color, becoming a stable species [34]. It is therefore used as an indicator of the antioxidant capacity of any substance that can donate hydrogen to the DPPH free radical. This assay is commonly used to estimate the antioxidant activity of a substance [33].

Samples were prepared as follows: 3 mL DPPH reagent solution of 1 μM concentration was mixed by shaking with different amounts of the solution to be analyzed (100 μL, 200 μL), after which the absorbances were measured minute by minute.

Absorbances were measured using a Rayleigh UV2601 UV/Vis Double Beam Spectrophotometer (Beijing Beifen-Ruili Analytical Instrument, Beijing, China).

### 2.3. Phyto-Homeopathic Product and Test Sample Preparation

Eye Blend Secom is a phyto-homeopathic product in tablet form, purchased from a specialist shop. Eye Blend is an innovative combination of extracts from 6 herbs and 5 homeopathic components. This blend is patented and contains the following plant products that have multiple active ingredients and important organic compounds:

- The aerial part of Silurium (*Euphrasia officinalis*), an herbaceous plant, found mainly in Europe, rich in glycosides, flavonoids, tannin, polyphenolcarboxylic acids (caffeic, ferulic), amino acids, vitamins (vitamin C, vitamin A), and minerals (silicon, iron, magnesium, phosphorus, potassium, zinc), having anti-inflammatory, cholagogue, hepatoprotective, and hypoglycemic effect, being recommended as an adjuvant in liver diseases, diabetes, eye diseases (conjunctivitis, inflammation of the lacrimal gland, ocular trauma, dystrophic diseases), respiratory diseases, and digestive diseases.
- The root of the Goldenseal plant (*Hydrastis Canadensis*) is considered a natural antibiotic due to its antibacterial, antifungal effect, and a protector of the body’s mucous membranes due to its alkaloid content with an immunostimulating, antispasmodic, sedative, hypotensive, tonic-uterine, choleretic and carminative effect.
- Dandelion root (*Taraxacum officinale*) which is rich in vitamins (A, B, C and D), proteins, and carbohydrates, with detoxifying, choleretic-cholagogues, diuretic, laxative effects, being recommended as an adjuvant in liver, digestive, cardiovascular and anemia diseases.
- Raspberry leaf (*Rubus idaeus*) containing pectins, organic acids (citric acid, malic acid), vitamins (vitamin A, B-complex, vitamin C, vitamin E), minerals (copper, calcium, iron, iodine, potassium), and tannin. This plant has antioxidant, diuretic, anti-inflammatory, astringent, carminative, depurative effects, and is recommended as an adjuvant in gynecological disorders, premenstrual syndrome, dysmenorrhea, menopause, gastrointestinal disorders, and respiratory disorders.
- Fennel fruit (*Foeniculum vulgare*) is rich in dietary fiber, vitamins (B complex, vitamin C), and minerals (calcium, iron, magnesium, phosphorus, potassium, zinc), with a carminative, antispasmodic, sedative, nervous tonic, expectorant, diuretic, hypotensive, and galactogenic effect, and is recommended as an adjuvant in gastrointestinal disorders, respiratory disorders, and depressive conditions.
- Ulm bark (*Ulmus rubra*) is rich in minerals (magnesium, calcium, iron, manganese, potassium, phosphorus), vitamins (vitamin B_1_, B_2_, B_3_, vitamin C), tannin, and phytosterols (beta-sitosterol, campesterol), with an emollient, expectorant, diuretic, anti-inflammatory, antioxidant, detoxifying, digestive effect, being recommended as an adjuvant in respiratory diseases, and gastrointestinal diseases (diarrhea, irritable bowel syndrome, indigestion, gastroesophageal reflux). The eye blend Secom is taken as 2 capsules twice a day with meals, and is contraindicated in bile duct blockage, acute cholecystitis, and/or intestinal obstruction.

For the preparation of the test sample, the capsule contents were weighed with the analytical balance (0.41 g) and then crushed in a pistil mill. The powder was added to a volume of 50 mL 0.1 M PBS solution, pH 7.0. The Elmasonic ultrasonic bath (Carl Roth GmbH, Karlsruhe, Germany) was required for complete dissolution and homogenization of the Eye Blend capsule contents. The sample was subjected to analysis by cyclic voltammetry using the CNF/SPE sensor.

### 2.4. Preliminary Studies for the Characterization of the Electrode

Preliminary studies characterizing the CNF/SPE sensor were carried out in both PBS solution at pH 7.0 and 10^−3^ M potassium ferrocyanide solution, and described in detail in a previous paper [31]. Following the calculations, the active surface area of the sensor had a value of 0.1819 cm^2^ [31], which was higher than the geometrical area, showing an optimal sensitivity of the sensor.

## 3. Results and Discussions

### 3.1. Voltammetric Behavior of CNF/SPE in Ferulic Acid and Caffeic Acid Solutions

In the next step, 10^−3^ M ferulic acid and 10^−3^ M caffeic acid solutions (PBS 10^−1^ M supporting electrolyte, pH 7.0) were analyzed with the CNF/SPE sensor using cyclic voltammetry.

**Figure 1:**
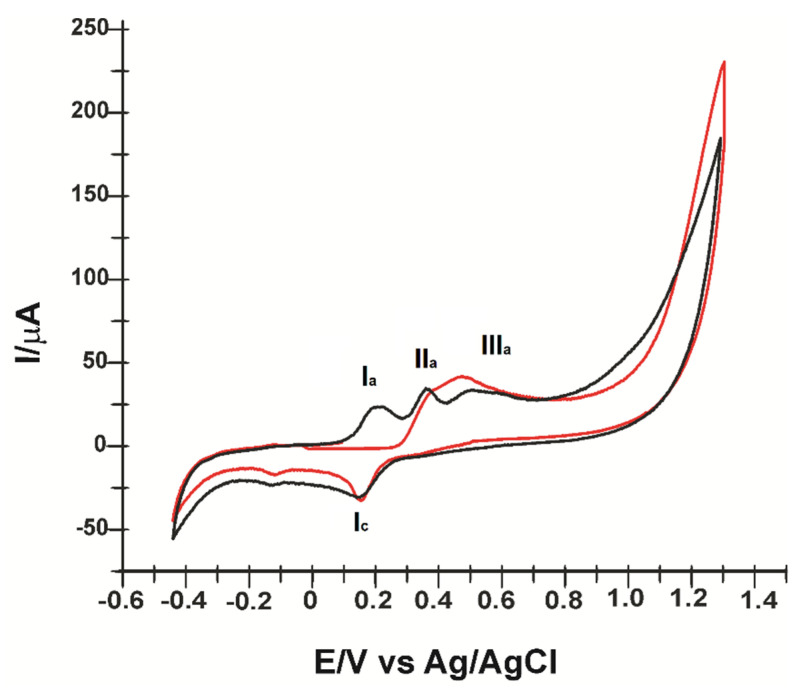
Cyclic voltammogram carbon nanofiber modified screen-printed electrode (CNF/SPE) immersed in 10^−3^ M ferulic acid solution and 10^−1^ M PBS at 0.1 V/s: first (red line) and second scan (black line).

Figure 1 shows the cyclic voltammogram obtained by CNF/SPE by immersion in 10^−3^ M ferulic acid solution (PBS electrolyte pH = 7.0) at the second scan. Three anodic peaks and two cathodic peaks can be seen well highlighted.

Initially, the recorded cyclic voltammogram showed two irreversible oxidation peaks, II_a_ at E = 0.388 V and III_a_ at E = 0.552 V, related to the beginning of the electropolymerization process. The measured potential value is close to that obtained in other studies [35]. At the second scan (Figure 1), the II_a_ and III_a_ peaks decrease in intensity and the peak pair, I_a_/I_c_, is highlighted, indicating the deposition of an electroactive film on the electrode surface [35].

The peak pair I_a_ (I_a_ = 23.308 μA, E = 0.235 V)/I_c_ (I_c_ = −23.87 μA, E = 0.179 V), corresponds to a quasi-reversible redox process (oxidation–reduction mechanism of the o-quinone/o-hydroquinone system). An additional II_c_ peak can also be observed without an anodic counterpart.

**Scheme 1:**
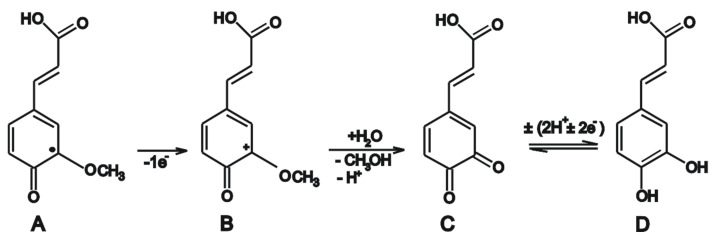
Electrochemical detection of caffeic acid. A—o-quinone of ferrulic acid; B— carbocation of ferrulic; C—3.4-dioxocinamic acid; D—caffeic acid. Adapted from [36].

Peaks II_a_ and III_a_ are related by the oxidation of FA, eliminating one electron and one proton, leading to a stable radical, followed by a second electron elimination, by cleavage of the methoxy group, leading to the formation of o-quinone (Scheme 1A), a reaction that explains the reversible I_c_/I_a_ system [35]. At this stage, caffeic acid could be formed, assuming that o-quinone (A) undergoes a second electron transfer leading to the formation of a carbocation (B), which is transformed by hydrolysis into 3.4-dioxocinamic acid (C) and a methanol molecule. During reverse scanning, 3,4-dioxocynamic acid is reduced to caffeic acid (D), which may correspond to peak III_a_ [36].

**Figure 2:**
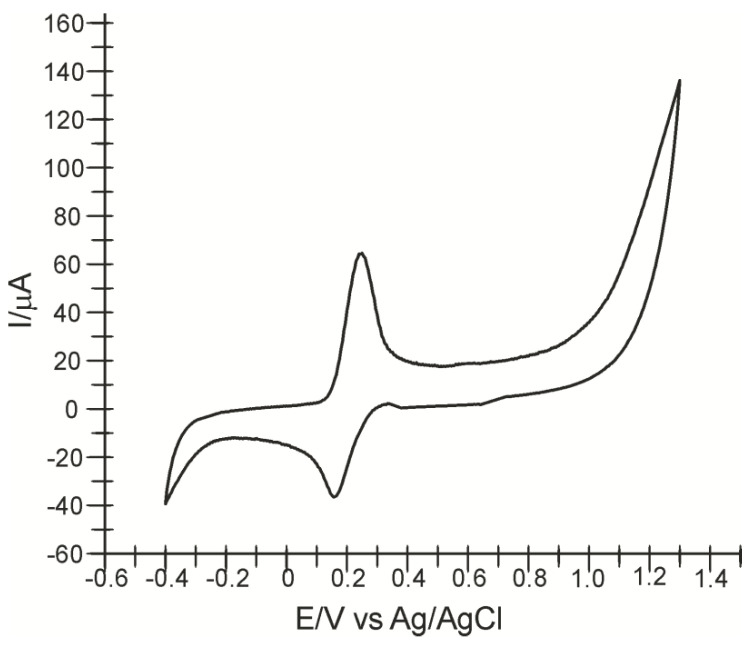
Cyclic voltammogram CNF/SPE immersed in 10^−3^ M caffeic acid solution (0.1 M PBS pH = 7.0) at 0.1 V·s^−1^.

The electrochemical behavior of CNF/SPE in a 10^−3^ M-PBS 0.1 M pH 7.0 caffeic acid solution was also studied. Figure 2 shows an anodic and a cathodic peak, related to the oxidation–reduction process of caffeic acid involving the transfer of two electrons and two protons.

The redox processes consist of oxidation of caffeic acid to the corresponding o-quinone derivative, followed by reduction of the o-quinone to the initial compound as mentioned in the literature. The electrodetection of caffeic acid using the CNF/SPE sensor involves the transfer of two electrons and two protons in one step [37,38,39].

**Scheme 2:**
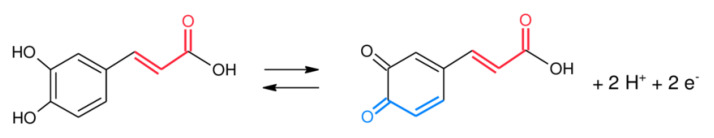
Mechanism of caffeic acid electrodetection.

This oxidation–reduction mechanism would be responsible for the effectiveness of caffeic acid as an antioxidant (Scheme 2) [40]. The anodic peak is recorded at E_a_ = 0.242 V with I_pa_ = 64.055 μA and the cathodic peak at E_c_ = 0.160 V and I_pc_ = −36.461 μA.

The mechanism of caffeic acid electrodetection is presented in Scheme 2.

The influence of the scan rate on the voltammetric response of the CNF/SPE sensor in CA solution, i.e., FA 10^−3^ M (0.1 M PBS pH 7.0) supporting electrolyte, at different scan rates between 0.1 V/s and 1.0 V/s, increasing the scan rate each time by 0.1 V/s, was further investigated.

**Figure 3:**
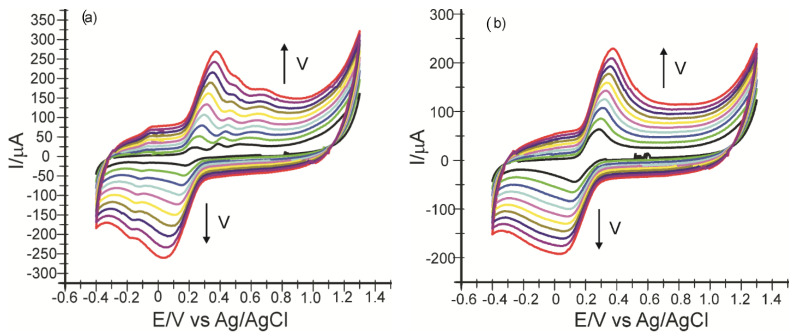
Cyclic voltammograms recorded, at different scan rates (0.1–1.0 V·s^−1^), of CNF/SPE immersed one at a time in 10^−3^ M ferulic acid solution (**a**) and 10^−3^ M caffeic acid solution (**b**).

Figure 3 shows the increase in the intensity of the anodic and cathodic peaks in both cases.

**Figure 4:**
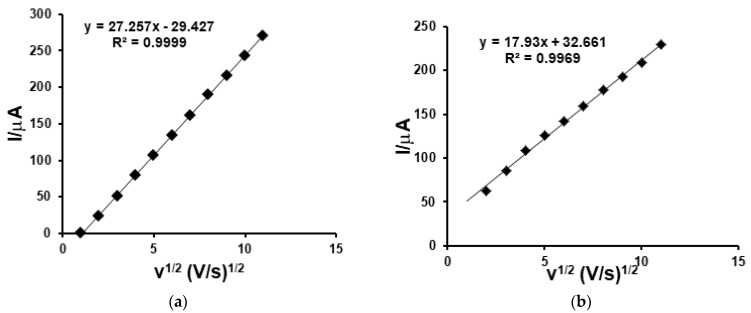
Dependence of cathode peak (I_p_) vs. scan rate obtained with CNF/SPE immersed in 10^−3^ M ferulic acid solution (**a**) and 10^−3^ M caffeic acid solution (**b**) in turn.

A graph of the anodic peak currents as a function of the square root of the scan rate showed a linear dependence between the two parameters, demonstrating that the redox process is controlled by the diffusion phenomenon (Figure 4).

Thus, for the calculation of the diffusion coefficient, we use the Randles–Sevcik equation, knowing from previous studies the active surface area of the CNF/SPE (0.1819 cm^2^) [38].

$$I_{pa}=268,600\times n^{3/2}\times A\times D^{1/2}\times Cv^{1/2}$$

The results obtained are presented in Table 1.

The diffusion coefficient values of CA and FA are lower than other values obtained in articles reported in the literature [36,41,42,43].

In addition, for caffeic acid, there is a slight difference between the value obtained now, compared to that obtained in the previous study (4.13 × 10^−6^ cm^2^·s^−1^) [33], although the same type of electrode was used. These differences in the value of D might be explained by the use of an electrolyte solution with a higher pH [44].

### 3.2. Influence of FA and CA Concentration on the Electrochemical Response

The following experiments aim at the simultaneous determination of the two hydroxycinnamic acids using CNF/SPE. For this purpose, solutions of 10^−3^ M concentration of CA and FA, respectively, were used in turn. For each case, a calibration curve was made for the other phenolic compound added in the 10–1000 μM concentration range.

**Figure 5:**
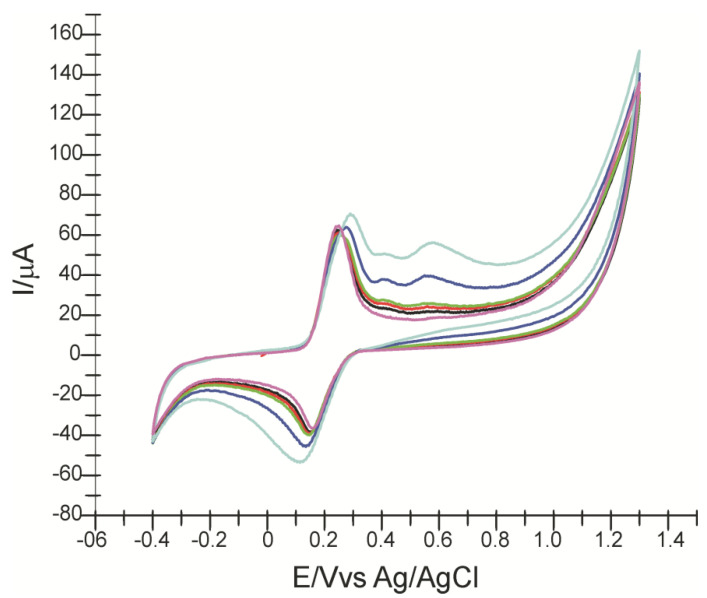
Cyclic voltammograms of CNF/SPE in 10^−3^ M caffeic acid solution (purple line), then containing different concentrations of ferulic acid: 10 μM (black line), 50 μM (red line), 100 μM (green line), 500 μM (blue line) and 1000 μM (light blue line).

Figure 5 shows the cyclic voltammograms obtained after immersion of CNF/SPE in a solution of 10^−3^ M caffeic acid (electrolyte support PBS pH = 7.0) which contains increasing concentrations of ferulic acid.

An anodic and a cathodic peak of higher intensities corresponding to the presence of caffeic acid can be observed, and as increasing amounts of ferulic acid are added, the irreversible II_a_ and III_a_ peaks corresponding to FA electropolymerization are better highlighted. FA deposition on the electrode surface corresponding to a pair of I_a_/I_c_ peaks overlaps with peaks corresponding to the oxidation–reduction process of CA.

Upon addition of the first FA concentrations, the first anodic peak undergoes a slight decrease in intensity, which means that the oxidation process of CA is inhibited by the oxidation and deposition of FA. In contrast, when the concentration of FA added to the solution is equal to the concentration of CA, both the intensity of the anodic peak (I_pa_ = 70.394 μA) and the potential (E_a_ = 0.287 V) display higher values. This can be related to the overlap of the oxidation waves of CA and FA, which have the close oxidation potentials [31,45].

Compared to the anodic peak, the cathodic peak increases in intensity in proportion to the increase of FA concentration in solution, concomitant with the shift of the potential towards lower values (la 1000 μM FA-E_c_ = 0.111 V). This shows that both compounds undergo a simultaneous reduction process.

**Figure 6:**
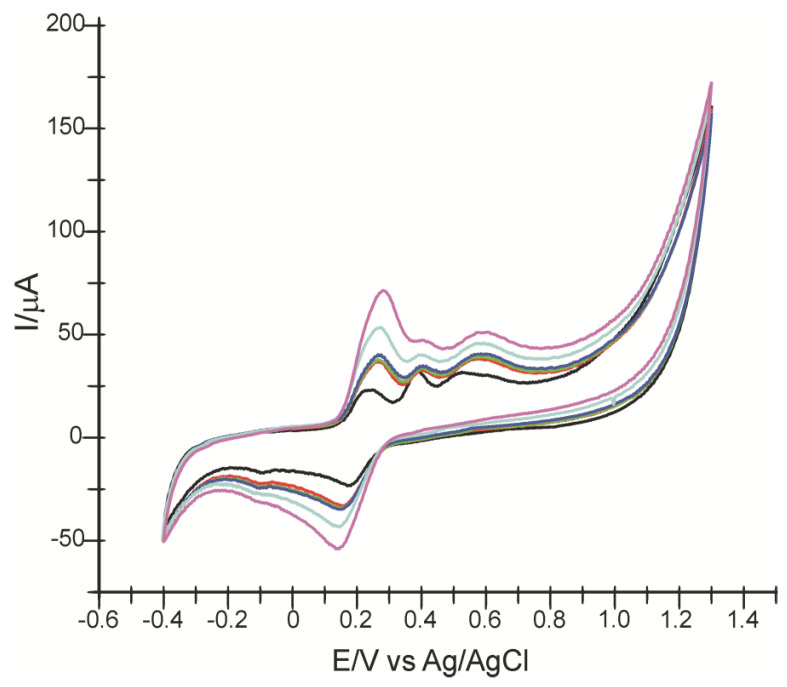
Cyclic voltammograms of CNF/SPE in FA 10^−3^ M solution (black line), then containing different CA concentrations: 10 μM (red line), 50 μM (green line), 100 μM (blue line), 500 μM (light blue line) and 1000 μM (purple line).

Figure 6 shows the cyclic voltammograms obtained from CNF/SPE recorded one at a time in a 10^−3^ M FA solution, concentration remaining constant, increasing the concentration of CA (10–1000 μM).

As the concentration of CA in the solution increases, a higher intensity of the first anodic peak, related to the oxidation of caffeic acid, is revealed. The presence of CA in the solution does not prevent the oxidation of FA. The constant increase in the intensity of the anodic peak is explained by the mechanism of caffeic acid formation following ferulic acid oxidation (as shown in Scheme 1), but also by the synergy of the oxidation processes of the two compounds.

As in Figure 6, when the same concentration of CA and FA is present in the solution, the potential of the first anodic peak shifts to a higher value. As for the cathodic peak, when in the solution with a constant concentration of FA, and amounts of CA are progressively added, the intensity of the peak increases at a faster rate and the potential shifts towards lower values.

Studies suggest that in the case of CA and FA, the reactions whereby electron and proton transfer take place are consecutive and occur under kinetic control of the chemical deprotonation reaction, due to the conjugation of the aromatic double bond ring which exerts a major stabilization of the cation generated from the two compounds [46].

Based on the cyclic voltammograms obtained above, the relationship between added concentrations and cathodic peak intensity for CA and FA was studied. The cathodic peaks had a constant increase in both situations, showing that the reduction process was influenced by the presence of both antioxidants. The concentration range studied was 10–1000 μM for the phenolic compound studied, while the concentration of the other was constant.

For the calibration of the sensor towards CA and FA, respectively, we subtracted the initial cathodic currents (in the presence of 10^−3^ M CA or FA) from the observed cathodic currents, after the additions of the CA or FA of different concentrations. The ΔI (μA) was calculated as:

ΔI = I_obs_ − I_ini_

where:

- I_obs_ is the observed cathodic current after the addition of analytes, μA;
- I_ini_ is the initial cathodic current before the addition of analytes, μA.

**Figure 7:**
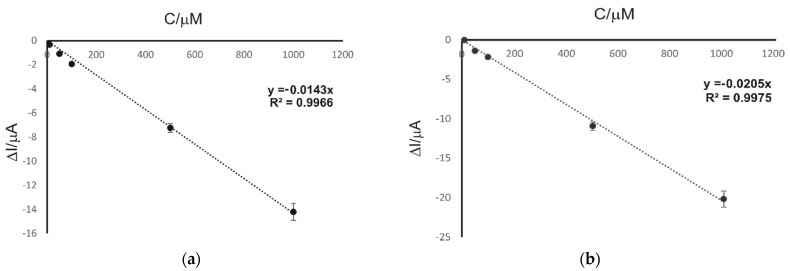
Linear dependence between ΔI and concentration of FA (**a**) and CA (**b**) based on voltammetric measurements with CNF/SPE (concentration range is 10–1000 μM).

The calibration linear models, ΔI vs. concentration, are presented in the Figure 7.

As can be seen in Figure 7, there is a linear dependence between the ΔI and the concentration of FA (a) and CA (b), respectively. The equations of the calibration lines and the limit of detection (LOD) and limit of quantification (LOQ) values obtained are noted in Table 2.

The low detection and quantification limit values demonstrate the increased sensitivity of the sensor to determine FA and CA simultaneously, and the feasibility of the voltammetric method used. The LOD and LOQ values obtained in the present study are similar to those presented in other studies [21,47,48] (Table 3).

### 3.3. Interference Studies

To investigate the various interferences on the simultaneous determination of CA and FA, vanillic acid, gallic acid and quercetin were added in turn to the solution. Initially, concentrations of 5 × 10^−4^ M of each interfering compound were added in turn. The signal changes were undetectable. Then, higher concentrations of each compound (10^−3^ M) were added to test the tolerance limit. Quercetin had the most influence on the appearance of the peaks, due to its similar chemical structure to CA and FA. The results are shown in Table 4.

### 3.4. Sensor Stability and Repeatability

**Figure 8:**
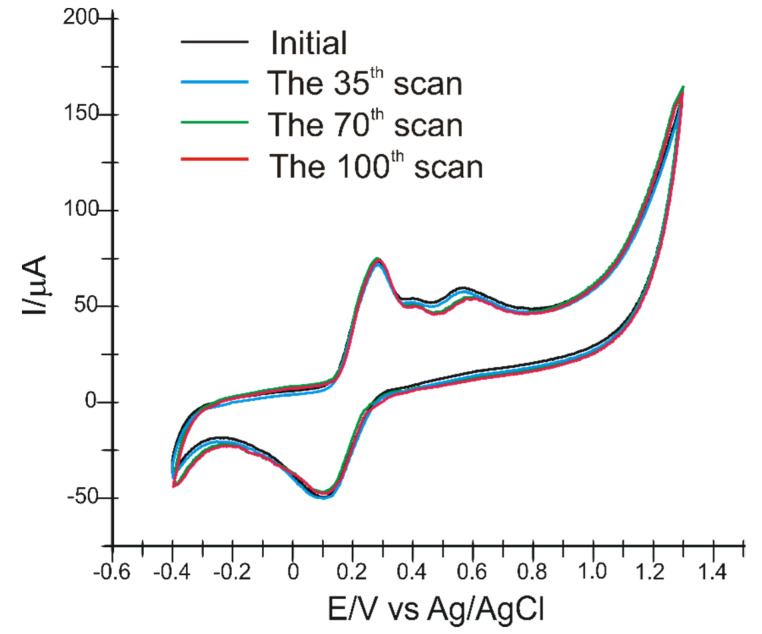
The repeatability of the sensor response under continuous use. Conditions: cyclic voltammetry, in solution of 10^−3^ M CA and FA (0.1 M PBS, pH 7.0).

To investigate the repeatability of the sensor, voltammetric measurements were performed in a solution of CA and FA (10^−3^ M of both compounds) with the same sensor 100 times. The results are presented in the Figure 8. The relative standard deviation (RSD) of the measurements was 4.5%. Between measurements, the CNF/C-SPE was rinsed with 0.01 M PBS, pH 7.0. Therefore, CNF/C-SPE can be used repeatedly.

**Figure 9:**
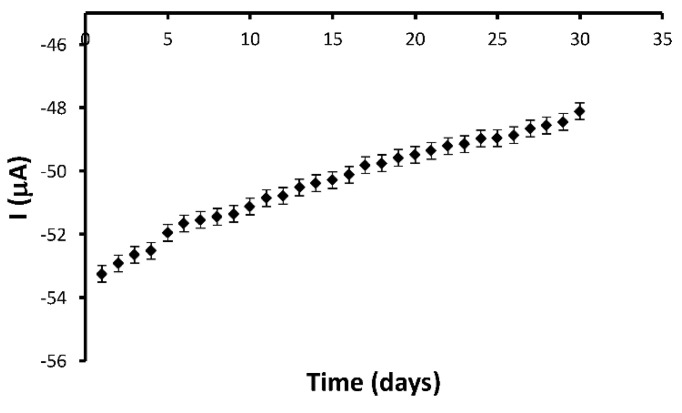
The long-term stability of the sensor response. Conditions: cyclic voltammetry measurements, 10^−3^ M CA and FA (in 0.1 M PBS, pH 7). The error bars indicate the standard error for three measurements with the same electrode.

The stability of the sensor was verified by monitoring the voltammetric response in a solution of equal concentration of CA and FA (10^−3^ M) at regular intervals (1 day) for a period of one month (Figure 9). The voltammetric data used in the stability studies was the cathodic peak current. Throughout this period, the sensor was stored at 4 °C in a refrigerator.

As can be observed in the Figure 9, the electrochemical response was maintained well, with the cathodic current being 90% from the initial current after one month.

### 3.5. Simultaneous Determination of CA and FA in the Eye Blend Product

**Figure 10:**
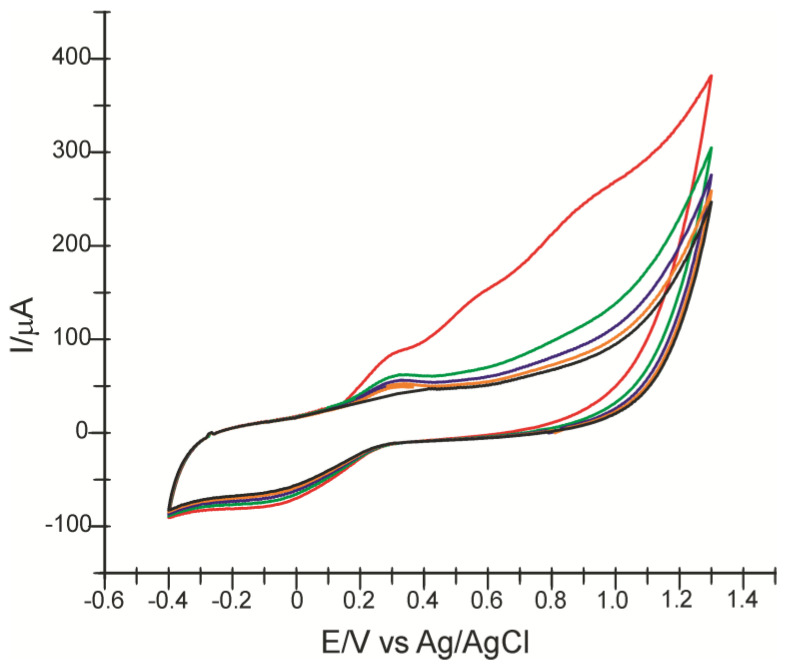
Cyclic voltammograms recorded with CNF/C-SPE immersed in Eye Blend solution (1 cps/50 mL PBS 0.1 M, pH 7.0): first scan—red line, second scan—green line, third scan—blue line, fourth scan—orange line, fifth scan—black line.

To confirm the feasibility of the method, the Eye Blend product was chosen to detect CA and FA in its composition using CNF/SPE. Figure 10 shows the first five CV scans recorded with CNF/SPE immersed in the solution obtained by dissolving Eye Blend capsules in 0.1 M PBS, pH 7.0.

The presence of ferulic acid is noted by recording the corresponding anodic peaks from the first CV scan. On subsequent scans, the second and third peaks decrease in intensity due to the deposition process of FA on the electrode surface [31]. The decrease in intensity of the two peaks could also be due to the presence of other electroactive compounds in the composition, which inhibit FA activity. The quantification of FA and CA was carried out using the cathodic peak, utilizing the values of the currents at the CA and FA characteristic potentials, respectively. The amounts of FA and CA were calculated using the calibration equations for each of the two compounds, and the results are shown in Table 5.

It can be argued that CA and FA are present in Eye Blend in similar amounts. The method developed in this study can be considered a cost-effective and easy-to-handle tool that could be applied to various other pharmaceutical products.

### 3.6. Determination of Antioxidant Capacity of CA and FA by DPPH Method and Correlation with Voltammetric Results

The DPPH method is based on the reaction with electron donors or hydrogen radicals (H▪) producing antioxidant compounds. The reduction of DPPH is directly proportional to the amount of antioxidant present in the sample being analyzed [32].

**Scheme 3:**
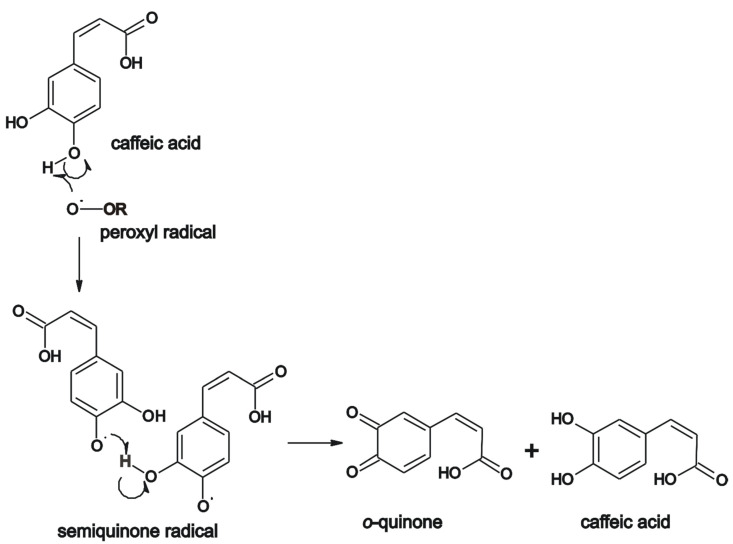
Mechanism of the antioxidant activity of caffeic acid. Adapted from [40,57].

The mechanism of the antioxidant activity of caffeic acid can be seen in Scheme 3.

In the first phase, the absorbance in DPPH was determined and the value of 0.935 was obtained. Subsequently, 100 μL, then 200 μL, of 10^−3^ M caffeic acid solution was added to the reagent solution. The same procedure was carried out for the 10^−3^ M ferulic acid solution. Between measurements, the mixture was stirred thoroughly.

It was observed that DPPH decolorization was dependent on the concentration of the phenolic compound in the sample. When equal concentrations of FA and CA (50 μL) were added to the DPPH solution, it was found that the reduction process of the reagent was more intense, resulting in a lower absorbance value. This shows that the presence of both phenolic compounds in the preparation (although in a lower concentration than when taken separately) favors a higher antioxidant effect.

For analysis of the real sample, the contents of one Eye Blend capsule were dispersed in 3 mL of chloroform. Then, 100 μL in 3 mL of DPPH reagent were added to this solution.

The radical scavenging activity was expressed as a percentage and was calculated using the following formula:

% R = (A_control_ − A_sample_)/A_control_ × 100

The results can be found in Table 6.

The results in Table 5 show that the antioxidant capacity of a product may be higher when both compounds (CA and FA) are found in the composition, due to the synergistic effect between them. However, in the mixture with lower concentrations of CA and FA, and a higher antioxidant capacity was determined (8.8206%).

The voltammetric responses of the sensor immersed in solutions with the same concentration, such as in the DDPH method, were recorded. Considering that the antioxidant capacity increases when both phenolic compounds are found in the solution, it is certified that in the voltammetric analysis, the cathodic current increases progressively with the increase of the concentration of CA and FA, respectively. This shows that the CNF/SPE sensor can be used to accurately quantify both phenolic compounds. The results of these two methods of analysis are well correlated. Moreover, voltammetric methods could be very useful for determining antioxidant capacity, and the DPPH method can be used to compare and validate electrochemical studies [58,59].

## 4. Conclusions

The main objective of the study was the simultaneous determination of two phenolic compounds, caffeic acid and ferulic acid, from a phyto-homeopathic formulation (Eye Blend) by means of cyclic voltammetry using a carbon nanofiber-based screen-printed sensor.

The sensor showed good sensitivity for the detection of both analytes in the solution to be analyzed. The novel element of the work is the study of the redox mechanism of each compound in the presence of the other. Subsequently, FA and CA were quantitatively determined from the Eye Blend product with CNF/C-SPE by cyclic voltammetry. Moreover, the influence of other species on the electrochemical signal was reduced, due to the sensor having favorable specificity. Subsequently, the antioxidant activity of the compounds was determined by the DPPH method, thus demonstrating the antioxidant, synergistic effect of the two phenolic compounds in the Eye Blend product.

Therefore, both the sensor and the voltammetric method used proved to be suitable for the simultaneous determination of the two phenolic compounds in a product with a complex composition, such as phenolic extracts of olive oils. It can be stated that this detection method is sensitive, accurate, easy to apply and could also be used for simultaneous determinations of other phenolic compounds.

## Figures and Tables

**Table 1:** Linear equations, R^2^ and diffusion coefficients of CA and FA.

Phenolic Compound	Linear Equation	R^2^	D (cm^2^·s^−1^)
CA	y = 1.793 × 10^−5^ − 3.266 × 10^−5^	0.9969	3.48 × 10^−6^
FA	y = 2.725 × 10^−5^ − 2.942 × 10^−5^	0.9999	7.24 × 10^−6^

**Table 2:** Limit of detection (LOD) and limit of quantification (LOQ) values, line equation and R^2^ of the CNF/SPE sensor for simultaneous detection of CA and FA.

CNF/SPE	LOD (M)	LOQ (M)	R^2^	Calibration Linear Equation
**FA detection**	2.33 × 10^−7^	7.78 × 10^−7^	0.9966	ΔI (μA) = −0.0143·c (μM)
**CA detection**	2.39 × 10^−7^	7.97 × 10^−7^	0.9975	ΔI (μA) = −0.0205·c (μM)

**Table 3:** Comparison of different methods to determine CA and FA.

Type of Sensor	Analyte	LOD (M)	LOQ	Reference
PGF/GCE	CA	1.25 × 10^−6^	-	[49]
GPC	CA	2.9 × 10^−7^	9.70 × 10^−7^	[50]
FLD-MWCNTs/SPE	CA	2 × 10^−7^	6.6 × 10^−7^	[51]
GCE-MoS_2_	CA	7.6 × 10^−7^	-	[52]
graphite-like carbon nitride (g-C3N4) and chitosan (CS)	CA	1.9 × 10^−7^		[21]
CNF/SPE	CA	2.39 × 10^−7^	7.97 × 10^−7^	This work
MWCNT modified GC	FA	1.17 × 10^−6^	-	[53]
DDAB/nafion/CPE	FA	3.9 × 10^−7^	-	[54]
Py-MWCNTs/GCE	FA	1.17 × 10^−6^	-	[55]
paper-based analytical device	FA	1 × 10^−6^	3 × 10^−6^	[56]
graphite-like carbon nitride (g-C_3_N_4_) and chitosan (CS)	FA	2.55 × 10^−6^	-	[21]
GN/GCE	FA	2 × 10^−7^	-	[47]
CNF/SPE	FA	2.33 × 10^−7^	7.78 × 10^−7^	This work

GPC—glassic polymeric carbon; PGF—poly(glutamic acid) film; FLD—functionalized larger diameter; MWCNTs—multi-walled carbon nanotubes; SPE—screen-printed electrode; GCE-MoS_2_—glass carbon electrode with flower-like MoS_2_ and PANI nanotubes; DDAB—didodecyldimethylammonium bromide; CPE—carbon paste electrode; Py-MWCNTs/GCE—polypyrrole-multiwalled carbon nanotubes modified glassy carbon electrode; GN/GCE—graphene modified glass carbon electrode.

**Table 4:** Interference of chemically related species on the simultaneous determination of CA and FA (10^−3^ M).

Interfering Compound	Ratio of Concentrations	Recovery/%	Ratio of Concentrations	Recovery/%
Quercetin	1:0.5	100 ± 4.6	1:1	96 ± 2.1
Vannilic acid	1:0.5	102 ± 1.9	1:1	98 ± 3.1
Gallic acid	1:0.5	102 ± 4.1	1:1	99 ± 3.5

**Table 5:** Quantitative data of the CA and FA determination in the Eye Blend product using CNF/C-SPE.

Phenolic Compound Detected	Voltammetric Method	
	mg/100 g	mg/Caps
**CA**	6.021	21.076
**FA**	7.516	26.306

**Table 6:** Determination of the antioxidant activity of CA and FA compounds.

Sample	3.22 × 10^−5^ M CA	6.25 × 10^−5^ M CA	3.22 × 10^−5^ M FA	6.25 × 10^−5^ M FA	1.63 × 10^−5^ M CA + 1.63 × 10^−5^ M FA	100 μL Eye Blend
% R	0.9625	1.5005	0.8658	1.8628	8.8206	12.7077

## Data Availability

Not applicable.
